# Positive Psychological Capital as a Predictor of Satisfaction With the Fly-In Fly-Out Model

**DOI:** 10.3389/fpsyg.2021.669524

**Published:** 2021-07-19

**Authors:** Nazaré Soares Marques, Miguel Pereira Lopes, Sónia P. Gonçalves

**Affiliations:** ^1^Escola de Ciências Económicas e das Organizações, Universidade Lusófona de Humanidades e Tecnologias, Lisboa, Portugal; ^2^Centro de Administração e Políticas Públicas, Instituto Superior de Ciências Sociais e Políticas, Universidade de Lisboa, Lisboa, Portugal

**Keywords:** FIFO, job satisfaction, micro-intervention, positive psychological capital, well-being

## Abstract

The flexibility of markets and international agreements have lured a growing number of companies to expand their business beyond frontiers in search for new markets and a bigger business network. Specifically, expatriates became keystones to implant and promote the so desired expansion into international markets, Particularly, Fly-in fly-out (FIFO) flexpatriates. Although FIFO work practices are widely used, little is known about how to promote these professionals’ perceived job satisfaction (JS) across the course of their work cycles. That is why the goal of our research is to test the positive psychological capital (PsyCap) applicability to Portuguese FIFO flexpatriates. In the midst of the positive psychology theories, Luthans et al. (2007b) underline that workers are the psychological capital of any organization. Therefore, the development of the PsyCap becomes crucial and also contributes to the promotion of JS, nowadays a construct intertwined with well-being. As such, we developed and applied a HERO–(hope, self-efficacy, resilience, and optimism)–micro-intervention in order to assess whether it moderated the relationship between a FIFO flexpatriates PsyCap and their JS. The research took place over three distinct moments, both PsyCap and JS were measured before and after the HERO micro-intervention, and again 3 months later. The data collected shows that a positive correlation exists between FIFO flexpatriates PsyCap and JS. Moreover, our results pointed out that the micro-intervention enhanced FIFO flexpatriates PsyCap, and also showed that this increase lasted over (at least) 3 months.

## Introduction

Fly-in fly-out is a model of international work applied to organizational flexpatriates with permanent residence in the country of origin; it is defined by frequent temporary journeys abroad (no more than 6 months) working for a company to perform management and/or formation jobs, to develop specific projects or to fill in the flexibility demands of the company (Torkington et al., 2011; Pini and Mayes, 2012; Brook et al., 2020).

However, the type of functions performed by FIFO flexpatriates and the constant distance from their families and friends’ raises concerns about health and safety, disturbances in social and family life, quality of work, effects on productivity performance, and job dissatisfaction. These workers have higher levels of stress, anxiety, and depression than the general population (Albrecht and Anglim, 2018; Center for Transformative Work Design (CTWD), 2018). Periods of medical discharge and frequent turnover, therefore, is considered to be in the best interest of companies to develop and apply strategies to promote the well-being of these workers. Organizations need to not only address the non-financial needs of their workers, but also to look to the development of their perceived support, job satisfaction (JS), and adjustment to the FIFO lifestyle (Brook et al., 2020). In practice, when an environment is challenging, people need additional resources (Basinska and Rozkwitalska, 2020).

In recent years it has become clear from the trends appearing in this literature that there are interventions that can help prevent widespread and escalating problems, intervention options that could assist once an issue arises, and intervention options for follow-up and improvements (Brook et al., 2020). Our study followed the theories of Fred Luthans et al., who combined theories of positivist psychology with the concepts of business management. In an increasingly competitive world the workers performance and workforce are the companies’ positive psychological capital (PsyCap). This mental potentiality was central in our study to understand the way in which company’s employees can develop the desirable positive psychological states and defining which techniques and methods work best when applied locally.

Positive psychological capital can be enhanced through the superior basic construct PsyCap that integrates four other constructs: hope, self-efficacy, resilience, and optimism (Luthans et al., 2015). The four constructs are considered to be state-like and, as such, capable of being developed (Luthans et al., 2007a) and altered. These four PsyCap constructs can contribute to an explanatory style through internalized perceptions of being in control. Each of the four capacities interacts with each other in order to create a unique way of acting (Luthans et al., 2007b).

According to Donaldson et al. (2020) positive organizational psychology interventions that target and improve hope, efficacy, resilience, and optimism (HERO) can be highly effective and a robust way to improve well-being and positive functioning at work across diverse geographical regions and cultures. In their study the authors found that PsyCap is strongly associated with workplace proactivity, proficiency, adaptivity, and overall work performance across 15 nations.

Following this, we assume that FIFO workers can see their resilience threatened by the loss of the social environment familiar to them; however, they can resort to hope in order to create new ways to face obstacles and rebuild their social relationships (Luthans et al., 2007b). Flexpatriates can enhance their resilience, analyzing the present unstable situation as being only temporary and/or transitory. This way of looking at the situation makes them aware of the need of some adjustment skills on their part for everything to go smoothly, which, in turn, will contribute to increase their resilience and performance, and leading to a new way of facing similar situations. Therefore, general well-being and JS are harnessed. Those who show a high PsyCap are flexible and adaptable to the changing needs of their jobs, while at the same time their PsyCap helps them maintain good levels of competence and well-being.

As PsyCap establishes itself in organizations, the question arises whether if the acquired skills are long lasting or deteriorate over time and requiring new interventions. We believe that PsyCap is a dynamic strategy in terms of creating resources to face the challenges imposed by the job requirements.

Studies carried out so far by different authors, in different environments and cultures, indicate that PsyCap can be used and adapted to cultural differences, so it can be used in the most diverse countries and cultures (Luthans et al., 2007b), which was verified in the Portuguese case.

In the same measure, and taking into account the same theories of positive psychology, we consider that JS is an essential factor for the well-being of workers and their good performance. It is also important for companies to take into account the degree of satisfaction of their workers in order to ensure a good performance in their functions, enhancing and optimizing the capacities of the business environment.

The present research intended to understand the relationship between the PsyCap and JS and more specifically understand if a micro-intervention to promote the employees PsyCap HERO (developing internal strategies to deal with and overcome adverse situations at a professional and personal level) would positively reflect on their JS degree. We used the guidelines provided by Luthans et al. (2007a), adapting the micro-intervention to the Portuguese organizational reality and to the specificity of FIFO demands.

The aim is to verify if this micro-formation method, already validated and tested in different professional contexts and particularly in an organizational environment, is also applicable to FIFO flexpatriates, contributing to JS and, subsequently, to the employees well-being in general.

To the purpose of this research, following Luthans and Youssef (2004), Luthans et al. (2007a), PsyCap means a positive psychological individual state regarding goals, defined by the self-efficacy to use the necessary effort, optimism about present and future success, hope to create pathways, and resilience to cope with obstacles and challenges. The definition of JS was constructed based on a broad approach to the classical definitions (Hoppock, 1935; Herzberg, 1964; Locke, 1969, 1976; Smith et al., 1969; Spector, 1985, 1997, 2006, 2012; Weiss and Brief, 2001)^1^ and the more recent studies on well-being (Diener et al., 1997^2^; Diener, 2000; Judge and Klinger, 2008^3^; Siqueira and Padovam, 2008; Diener et al., 2009; Youssef-Morgan and Luthans, 2015^4^). JS is an individual emotional and cognitive evaluation regarding all aspects concerning the person’s job that reflects itself on the person’s attitudes toward the job and company and on the general well-being of the person.

## Materials and Methods

### Participants

A total of 143 Portuguese FIFO flexpatriate workers from two multinational companies (energy/IT and lifts/freight elevators), with a shared parent country cultural background and working in partnership to operate in complementary industrial areas, were randomly divided into two groups: control group (CG) with 75 participants and experimental group (EG) with 68 participants. The two groups were identical (see Table 1).

**TABLE 1:** Sociodemographic characteristics of participants at baseline.

	CG (*n* = 75% and 52.4%)		EG (*n* = 68% and 47.6%)	
	N	%	N	%
**Gender**				
Male	54	72.0	58	85.3
Female	21	28.0	10	14.7

Total	75	100.0	68	100.0

**Age**				
30 years or less	5	6.7	9	13.2
Between 31 and 40 years	32	42.7	32	47.1
Between 41 and 50 years	24	32.0	17	25.0
51 years or more	14	18.7	10	14.7

Total	75	100.0	68	100.0

**Marital status**				
Single	23	30.7	13	19.1
Married	30	40.0	36	52.9
Divorced	12	16.0	5	7.4
Registered partnership	10	13.3	14	20.6

Total	75	100.0	68	100.0

**Education**				
Graduation	15	20.0	12	17.6
Specialization	31	41.3	12	17.6
Post-graduation	8	10.7	5	7.4
MA	10	13.3	8	11.8
Other	11	14.7	31	45.6

Total	75	100.0	68	100.0

**Job**				
Director	9	12.0	4	5.9
Advanced engineer	3	4.0	2	3.5
Engineer/Level A Specialist/Supervisor	17	22.7	20	29.4
Manager/Business Manager/Specialist/Technician	24	32.0	9	13.2
Maintenance technician	17	22.7	33	48.5
Assistant/Clerk	5	6.7	0	0.0

Total	75	100.0	68	100.0

**Years working for the organization**				
5 years or less	13	17.3	11	16.8
Between 6 and 10 years	20	26.7	23	30.1
Between 11 and 15 years	15	20.0	14	20.3
16 years or more	27	36.0	20	32.9

Total	75	100.0	68	100.0

**Years on current job**				
5 years or less	19	25.3	17	25.0
Between 6 and 10 years	27	36.0	29	42.6
11 years or more	29	38.7	22	32.4

Total	75	100.0	68	100.0

**Years under FIFO**				
5 years or less	21	28.0	16	23.5
Between 6 and 15 years	28	37.3	32	47.1
16 years or more	26	34.7	20	29.4

Total	75	100.0	68	100.0

### Methodology

This study is of a quantitative nature (Quivy and Campenhoudt, 1992), specifically the study was designed using a quasi-experimental longitudinal methodology. It intended to answer the question. Can a micro intervention influence the relationship between PsyCap and JS? In order to answer this question three hypotheses were developed.

#### Hypothesis 1

PsyCap positively influences JS of FIFO flexpatriates.

#### Hypothesis 2

A HERO micro intervention moderates the relation between a FIFO flexpatriate PsyCap and JS PSYCAP.

#### Hypothesis 3

The positive influence of the HERO micro intervention to increase the FIFO flexpatriate PsyCap lasts, at least, for 3 months.

**FIGURE 1:**
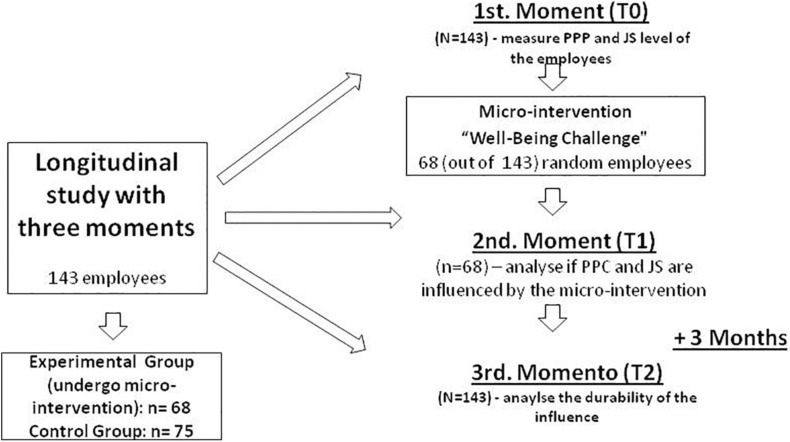
Experimental plan.

To corroborate them, we followed the experimental plan depicted on the procedure section (see Figure 1).

### Measurement

#### In Addition to the Basic Characterization Variables

Demographic characteristics (gender, age, marital status, education, and job), job description (years working in the company and at a specific job) and specific FIFO characteristics (years working as flexpatriate and time spent away from home)–the following two variables were also assessed.

##### Job satisfaction survey

Job satisfaction was assessed using the JS Survey developed by Spector (1985), which is a 36-item on a 6-point scale survey measuring 9 job dimensions and global JS. The coefficient alpha for global scale was 0.98 and 0.88 for salary, 0.90 for promotion, 0.90 for supervision, 0.84 for fringe benefits, 0.88 for contingency rewards, 0.89 for colleagues, and 0.90 for job nature.

##### PsyCap questionnnaire

The PsyCap Questionnaire developed by Luthans et al. (2007a) consists of 24 items on a 6-point scale to assess hope, self-efficacy, optimism, and resilience. The coefficient alpha for global scale was 0.98. Subscales values are 0.97 for self-efficacy, 0.96 for hope, 0.94 for resilience, and 0.95 for optimism.

##### Normality

The normality of the data distribution was analyzed using the kolmogorov-smirnov (KS) test, which is based on the comparison of the expected values (referred to as theoretical distribution) with the observed values (referred to as empirical distribution), and for equal or greater samples to 30. This test not only reveals a high sensitivity to the sample size, but it also systematically indicates the rejection of the hypothesis of normal distribution (Martinez and Ferreira, 2007).

One of the proposals found in the literature to overcome this situation is the use of the central limit theorem, which consists of two processes of division. On the one hand, the value of the skewness coefficient (Skewness) is divided by its standard error (Standard Error of Skewness). On the other hand, the value of the kurtosis coefficient (Kurtosis) is also divided by its standard error (Standard Error of Kurtosis). When the results of these processes are within the reference range [-1.96; 1.96], we can say that the distribution is approximately normal.

A detailed analysis of the distribution of the data that make up the global scales, as well as the dimensions that compose them, shows that the coefficients of asymmetry and kurtosis are within the reference ranges for an approximately normal distribution (see Table 2).

**TABLE 2:** Asymmetry and kurtosis coefficients (global scales and subscales).

Dimensions	KS	CS	CK
PSYCAP (global scale)	0.130**	1.15	*−*1.31
Self-efficacy	0.161**	1.26	*−*1.86
Hope	0.145**	1.10	*−*1.89
Resilience	0.125**	1.31	*−*1.87
Optimism	0.129**	1.31	*−*1.67

JS (global scale)	0.081*	1.95	*−*1.45
Payment	0.109**	1.40	*−*0.59
Specials	0.125**	1.20	*−*1.17
Supervision	0.115**	0.83	*−*1.78
*Fringe Benefits*	0.119**	1.27	*−*1.00
Contingent rewards	0.120**	1.92	*−*0.49
Operating conditions	0.146**	1.49	*−*0.56
Contributors	0.107**	1.38	*−*1.83
Nature of work	0.114**	1.36	*−*1.95
Communication	0.118**	1.62	*−*0.85

* *p* < 0.05; ** *p* < 0.001; KS, *Kolmogorov-Smirnov*; CS, *Skewness* coefficient; CK, *Kurtosis* coefficient.

#### FIFO Flexpatriates Satisfaction

Furthermore, two specific FIFO questions were contemplated in this study. These regarded their level of satisfaction with frequent journeys and their level of satisfaction with nights spent abroad.

### Procedure

The research was developed in three distinct moments. All participants completed the pre-test survey (moment 1 = T0) and then the participants assigned to the EG completed a 3-h micro-intervention (see Table 3) that aimed at for an increase in FIFO flexpatriates PsyCap. Prior to the intervention, the importance of positive and negative emotions on job performance and satisfaction were explained, as well as the goal of the intervention. After the intervention, all the participants assigned to EG completed the post-test survey (moment 2 = T1). 3 months later, all participants (EG and CG) completed the survey again (moment 3 = T2). These procedures are depicted in Figure 1.

**TABLE 3:** Micro-intervention “Well-Being Challenge.”

Steps	Tools
Introduction	• Promote well-being • Promote job performance and satisfaction
Goal definition	• Think SMART • Individual goals • Stepping
Group discussion	• Critical thinking • Alternative pathways
Past success	• Agency • Relevant models • Persuasion and positive feedback
Managing tasks	• Reinforcement of positive traits • Overcoming of personal shortcomings • Understanding the role of fear and illusion
Sum of the session so far	• Critical thinking and consolidation of self-efficacy, hope and optimism strategies
Steps of the way	• Foreseeing, preventing and overcoming obstacles

**Intermission**	

Possible scenarios	• Brainstorming • Agency • Using HERO to succeed
Conclusion	• What have we learned? • What tools have been acquired?

### Statistical Analyses

Statistical analyses using SPSS (descriptive analysis and inferential analysis) were performed. To test Hypothesis 1, PsyCap positively influences JS of FIFO flexpatriates, Pearson Correlations were carried out. To test Hypothesis 2, A HERO micro intervention positively moderates the relation between a FIFO flexpatriate PsyCap and JS, regression analyses were performed to test the moderating effect of the intervention on the relationship between PsyCap and JS. Finally, to test Hypothesis 3, the positive influence of the HERO micro intervention to increase the FIFO flexpatriate PsyCap lasts, at least, for 3 months, a *t*- test was performed for paired samples that aimed to assess the behavior of employees in two distinct moments.

## Results

The data collected through the instruments described above were analyzed with IBM’s Statistical Package (SPSS) and produced the following results.

### FIFO Flexpatriates PsyCap Influence on Job Satisfaction

The association between the variables under study is shown in Table 4 for all testing moments.

**TABLE 4:** Correlations for study variables.

	1	2	3	4	5	6
(1) PSYCAP_T0	–					
(2) JS_T0	0.876**	–				
(3) PSYCAP_T1	0.452**	0.415**	–			
(4) JS_T1	−0.220	0.021	0.380**	–		
(5) PSYCAP_T2	0.277*	0.251*	0.726**	0.395**	–	
(6) JS_T2	−0.036	0.209	0.269*	0.679**	0.520**	–

Note: **p < 0:01, *p < 0:05.

The correlations between PsyCap and JS, at T0 (before HERO), suggest that the higher the PsyCap levels, the more satisfied the participants are with their job (*r* = 0.876, *p* < 0.001). This association continues at T1 (after HERO), despite being weaker (*r* = 0.380, *p* < 0.001), and intensifies again (*r* = 0.520, *p* < 0.001) at T2 (3 months after the IM).

### A HERO Micro Intervention Moderates the Relation Between a FIFO Flexpatriate PsyCap and Job Satisfaction

In order to test the second hypothesis, it is important to know the moderating role of the HERO intervention performed, in the relationship between the PsyCap and the JS. To this end, the moderating variable was transformed into a dummy (0 = No and 1 = Yes), with 1 being the reference category.

To facilitate the interpretation of the data, the CPP and ST were recoded according to the midpoint of the scale, culminating in three reference points. Thus, it was considered that the moderate level of PsyCap corresponds to the value of the mean (*M* = 4.82; *SD* = 0.44), more or less a standard deviation [4.38 to 5.26], the low level oscillates between 1 and 4.37, and the between 5.27 and 6.0. The same procedure was followed for JS (*M* = 4.31; *SD* = 0.48), with the following values being obtained: low satisfaction varies between 1 and 3.82; moderate satisfaction between 3.83 and 4.79; and high satisfaction between 4.80 and 6.0.

The linear model explains 48.8% (adjusted *R*^2^ = 0.488, *p* < 0.001) of the variation in the JS level, the same being significant [*F*(3,139) = 46,177, *p* < 0.001]. The significant interaction effect (*t* = −2,556, *p* < 0.001), points to the existence of moderation. In addition to this finding, we can say that moderation reveals a negative effect (*B* = −0.423) on JS. Consequently, it is concluded that the intensity of the relationship between the constructs decreases in the participants who did not attend the HERO micro intervention.

**FIGURE 2:**
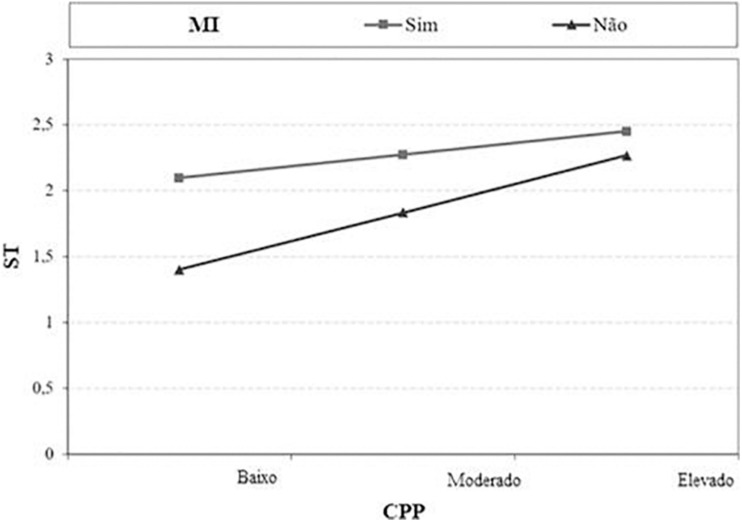
Correlation between micro-intervention, PPC, and JS. Ml, micro-intervention; ST, job satisfaction; CPP, PPC Sim = Yes; Não, no; Baixo, low; Moderado, moderate; Elevado, high.

The interaction effect is negative, because when employees do not attend HERO, the effect of PsyCap on JS is smaller. It was also possible to verify that PsyCap (β = 0.630, *t* = 7,243, *p* < 0.001) has a significant effect on JS, both in the CG and in the EG. However, for the employees who were the target of HERO (GE), this effect is bigger. The results obtained also demonstrate that HERO also has a positive impact on JS (β = 0.438, *t* = 4,515, and *p* < 0.001) (see Figure 2).

In view of the above, we can conclude that, after the HERO micro intervention, the levels of PsyCap and JS differ significantly depending on the group to which the participants belong, with the mean values of the EG being higher than those of the CG. The results also reveal the existence of a positive association between the PsyCap and JS. It is also concluded that the HERO micro intervention performed moderates the relationship between the PsyCap and the JS.

### Duration of the Positive Influence of the HERO Micro Intervention on FIFO Flexpatriates PsyCap and Job Satisfaction

In view of the above and taking into account the goals of this study, it was important to assess whether the PsyCap and JS of the participants belonging to the EG increased over the various moments of evaluation. For this purpose, we used paired samples *t*-tests.

The results obtained, which can be analyzed in Table 5, make it evident that there are statistically significant differences in the PsyCap [T0: *t*(67) = −9,656, *p* < 0.001; T1: *t*(67) = −10,671, *p* < 0.001; T2: *t*(67) = −5,607, *p* < 0.001] and in JS [T0: *t*(67) = −10,595, *p* < 0.001; T1: *t*(67) = −12,323, *p* < 0.001; T2: *t*(67) = −2.145, *p* < 0.05] between all the evaluated moments. Furthermore, it is possible to notice that from one moment to the next the average values increased for both variables. FIFO flexpatriates satisfaction with frequent journeys and nights spent abroad.

**TABLE 5:** Fly-in fly-out flexpatriates mean and standard deviation over time.

	EG						CG			
	T0		T1		T2		T0		T2	
	*M*	*SD*	*M*	*SD*	*M*	*SD*	*M*	*SD*	*A*	*SD*
Positive psychological capital (global)	3.51	1.04	4.60	0.43	4.82	0.44	3.46	0.95	3.37	0.96
Job satisfaction (global)	3.11	0.74	4.21	0.43	4.31	0.48	3.33	0.84	3.28	0.82

Comparing the results obtained on the first and third moments (see Table 6), we understand that the levels of satisfaction of the EG rise significantly from the first moment (before the micro-intervention) to the third moment (3 months after the micro-intervention). On the contrary, the CG similar results in both moments (slightly lower values, statistically not relevant).

**TABLE 6:** Satisfaction with journeys and nights spent abroad.

	EG		CG			
	*M*	*DP*	*M*	*DP*	*t-test*	*Sig.*
**T0**						
Satisfaction with journeys	3.32	0.93	3.21	1.09	−0.643	0.521
Satisfaction with nights spent abroad	3.13	1.12	3.15	1.04	0.078	0.938

**T2**						

	**EG**		**CG**			
	***M***	***DP***	***M***	***DP***	***t-test***	***Sig.***

Satisfaction with journeys	4.32	0.76	3.12	1.06	−7.640	0.000**
Satisfaction with nights spent abroad	4.34	0.80	3.10	1.05	−7.819	0.000**

Note: **p < 0:01.

## Discussion

Concerning the PSYCAP influence on the degree of JS of flexpatriates under FIFO the study shows that the higher the levels of PSYCAP, the higher the degree of JS of workers. This is evident, with different correlation levels, in all moments of research. The positive correlation between PsyCap and JS is stronger within the EG and its influence decays more rapidly on the flexpatriates included on the CG not subjected to the micro-intervention. These results are in conformity with the studies developed by Luthans et al. (2004, 2006,2007a,b, 2008, 2010, 2015), Luthans and Youssef-Morgan (2017), Lucas et al. (2014), and Alexandre (2017). The research conducted by Serrão (2018) also concluded a positive influence in the work environment, although not specifically with JS. Consequently, our study disproves many researchers (Côté and Morgan, 2002) who doubt the relationship between PsyCap and JS.

We ascertained that the micro-intervention has a moderation function between PsyCap and JS. The variation on the degree of JS demonstrated by both groups (EG and CG) shows that the members of the EG who completed the micro-intervention reveal a stronger correlation between PsyCap and JS and a higher degree of JS. After the micro-intervention, the average values increase for the global scales of PsyCap and JS, but also for the majority of subscales on both constructs. The studies conducted by Dello Russo and Stoykova (2015), Lucas (2015), and Neves (2016) reached the same conclusions.

Therefore, we determined that the effect of PsyCap improvement on JS is highly influenced by the micro-intervention. On the first moment of research, the average values on both groups are consistent. Nevertheless, on the second moment, after the intervention, the differences between both groups are significant. The average values of the EC are higher than those of the CG, confirming the validity and importance of these micro-interventions on the organizations. The results obtained show the validity of these micro-interventions as a useful tool for Human Resources in the Portuguese culture and professional environments, as also attested by the studies of Rego et al. (2010) and Antunes et al. (2017).

The positive influence of the micro-intervention on JS lasts at least 3 months, as proved by the survey made on the third moment of research; Dello Russo and Stoykova (2015) have already suggested this trend. The same is true concerning specific aspects of FIFO work. The participants assigned to the EG reveal a higher degree of JS with the journeys and nights spent abroad than the participants included in the CG.

The higher PsyCaps are self-efficacy and hope on the first moment, but on the third moment is resilience. The most valued JS dimension in all moments is supervision, although colleagues, and communication and work nature are also relevant to the participants. The PsyCap with the most impact on JS on the second moment is resilience and 3 months later (third moment) is optimism.

## Limitations

This kind of study always has a number of limitations regarding the sample and its representativeness and homogeneity. Although we try to diminish this situation, the sample we depend on was acquired in the two companies where we developed the research. Their representativeness is, of course, questionable and impossible to put to a strength test because there are no available data in Portugal considering flexpatriates. However, we try to guarantee certain homogeneity of both the experimental and CGs, although, also in this area, we are once again limited to the companies’ availability.

Also, it is recognized that the size of the present sample may have limited some of the analyses that we intended to conduct. For this reason, it is recommended that future studies include larger samples and consider the differences between individuals, for example with regard to age and professional function.

Considering that both PsyCap and JS involve subjective factors, such as personality traits and psychological characteristics, it would have been relevant to compare the results of the quantitative study with a qualitative approach. However, as this was not essential for the proposed objectives, we chose not to take this path.

## Implications

The objective of this research has always had in mind the JS and well-being of corporate employees inserted in the specific international work regime that we conceptualize as FIFO. In this sense, more important were the results obtained regarding flexpatriates in particular, which have implications for the health promotion and well-being and proved to be very promising regarding the use of this type of micro-interventions to improve, among other factors, the daily professional and personal life of employees covered by FIFO.

Our findings have several implications regarding the use of micro-interventions in companies to validate and value their PsyCap, this is, in fact, the added value and advantage they have to face today’s extremely competitive and constantly changing market.

This study is part of a broader panorama of interest in promoting and increasing PsyCap in the professional sphere, promoting health in the workplace and, simultaneously, in the most recent desire to study the specificities of international work and the FIFO work model in particular, contributing to systematically compile the range of coverage it has and the different types of work it can encompass within its specific work model.

Finally, we highlight the link that can be established between PsyCap and JS. The present study clearly reveals a relationship between both constructs and, in our view, the fundamental importance of developing both simultaneously.

## Conclusion

All evidence collected and analyzed suggests that the answer to the key question that guided the first steps of our work is that the improvement of the PsyCap through the promotion of micro-interventions positively influences the level of JS of the flexpatriates with their jobs in general and with the considered specificities of the FIFO job model. We were able to corroborate our first hypothesis, providing evidence that the PsyCap is a predictor of JS. The second hypothesis was also corroborated, because the medium levels of both global scales of PsyCap and JS increase with the experimental group after the micro-intervention, whereas the same levels remain similar within the CG (without micro-intervention. At last, the data collected on the third moment corroborated the third hypothesis as well, considering that the positive influence of the micro-intervention lasted at least 3 months. Although a lot is yet to be investigated, our study contributed with data to support the importance of micro-interventions to improve the PsyCap and we developed an instrument for the Human Resources Management to apply with international job models, specifically the FIFO model, in the organizational environment in Portugal.

Future studies may not only confirm our findings, but also use them to improve and deepen the content of micro-intervention, dedicating them specifically to FIFO, a work model whose negative psychological effects have not yet been fully investigated and deepened. We opted for the experimental method to fully enjoy the benefits in control and producing specific, relevant, and consistent results, ensuring the possibility of their replication and comparison with other studies that we hope may follow our effort.

## Data Availability Statement

The original contributions presented in the study are included in the article/supplementary material, further inquiries can be directed to the corresponding author.

## Ethics Statement

This study was carried out in accordance with the recommendations of ISCSP University with informed consent from all subjects.

## Author Contributions

NS performed the study and drafted the manuscript, under orientation by ML and SG. SG prepared the SPSS datasets analyzed by NS. All authors contributed to the article and approved the submitted version.

## Conflict of Interest

The authors declare that the research was conducted in the absence of any commercial or financial relationships that could be construed as a potential conflict of interest.

## Footnotes

1 Consulted in Judge and Locke (1993), MacDonald and Maclntyrem (1997), Santos and Mourão (2011), Guedes (2012), Spector (2012), Dalal (2013), Brenke (2015), Souza et al. (2015), and Sibhoko (2017).

2 Consulted in Youssef-Morgan and Luthans (2015).

3 Consulted in Siqueira and Padovam (2008).

4 Consulted in Arcidiacono and Martino (2016).

## References

[B1] Alexandre N. C. (2017). *O impacto da felicidade e do capital psicológico positivo nas atitudes e nos comportamentos dos colaboradores*. [Unpublished master dissertation]. Politécnico de Leiria, Leiria.

[B2] Albrecht S. L. Anglim J. (2018). Employee engagement and emotional exhaustion of fly-in-fly-out workers: a diary study. *Austr. J. Psychol.* 70 66–75. 10.1111/ajpy.12155

[B3] Antunes A. C. Caetano A. Cunha M. P. (2017). Reliability and construct validity of the portuguese version of the psychological capital questionnaire. *Psychol. Rep.* 7 1–17. 10.17575/rpsicol.v33i2.1421 28558609

[B4] Arcidiacono C. Martino S. D. (2016). A critical analysis of happiness and well-being. Where we stand now, where we need to go. *Commun. Psychol. Glob. Perspect. CPGP* 2 6–35.

[B5] Basinska B. A. Rozkwitalska M. (2020). Psychological capital and happiness at work: the mediating role of employee thriving in multinational corporations. *Curr. Psychol.* 2 1–14. 10.1007/s12144-019-00598-y

[B6] Brenke K. (2015). The vast majority of employees in Germany are satisfied with their jobs. *DIW Econom. Bull.* 5 429–436.

[B7] Brook E. Freeman M. Ditchburn G. (2020). The impact of fly-in, fly-out (FIFO) on the health and well-being of employees: what organisations can do to mitigate the risks and improve outcomes. *APPEA J.* 60 397–402. 10.1071/aj19146

[B8] Center for Transformative Work Design (CTWD) (2018). *Code of Practice: Mentally Healthy Workplaces for Fly-in Fly-out (FIFO) Workers in the Resources and Construction Sectors. Review Submission by the Centre for Transformative Work Design at the University of Western Australia. April 2018.* Perth, WA: University of Western Australia.

[B9] Côté S. Morgan L. (2002). A longitudinal analysis of the association between emotion regulation, job satisfaction, and intentions to quit. *J. Organ. Behav.* 23 947–962. 10.1002/job.174

[B10] Dalal R. S. (2013). *Job Attitudes: Cognition and Affect. In Weiner, I.B. Handbook of Psychology.* Hoboken, NJ: John Wiley & Sons Inc. 10.1002/9781118133880.hop212014

[B11] Dello Russo S. Stoykova P. (2015). Psychological capital intervention (PCI): a replication and extension. *Hum. Resour. Dev. Q.* 26 329–347. 10.1002/hrdq.21212

[B12] Diener E. (2000). Subjective well-being: The science of happiness and a proposal for a national index. *Am. Psychol.* 55, 34–43. 11392863

[B13] Diener E. Scollon C. N. Lucas R. E. (2009). “The evolving concept of subjective well-being: The multifaceted nature of happiness,” *Assessing Well-Being: The Collected Works of Ed Diener*, ed. Diener E. (Berlin: Springer Science), 67–100.

[B14] Diener E. Suh E. Oishi S. (1997). Recent findings on subjective well-being. *Indian J. Clin. Psychol.* 24, 25–41.

[B15] Donaldson S. I. Chan L. B. Villalobos J. Chen C. L. (2020). The generalizability of HERO across 15 nations: positive psychological capital (PsyCap) beyond the US and Other WEIRD Countries. *Int. J. Environ. Res. Public Health* 17:9432. 10.3390/ijerph17249432 33339210 PMC7765579

[B16] Guedes B. M. (2012). *Stresse em Expatriados – Transpor as Fronteiras de si.* Master thesis. Lisboa: Universidade Lusófona de Humanidades e Tecnologias.

[B17] Herzberg F. (1964). The motivation-hygiene concept and problems of manpower. *Pers. Admin.* 27, 3–7.

[B18] Hoppock R. (1935). *Job Satisfaction*. New York: Harper.

[B19] Judge T. A. Klinger R. (2008). “Job satisfaction: Subjective well-being at work,” in *The Science of Subjective Well-Being*, eds Eid M. Larsen R. J. (New York, NY: Guilford Press), 393–413.

[B20] Judge T. A. Locke E. (1993). Effect of dysfunctional thought processes on subjective well-being and job satisfaction. *J. Appl. Psychol.* 78 475–490. 10.1037/0021-9010.78.3.475

[B21] Locke E. A. (1969). What is job satisfaction? *Organ. Behav. Hum. Decis. Process* 4, 309–336.

[B22] Locke E. A. (1976). “The nature and causes of job satisfaction,” in *Handbook of Industrial and Organizational Psychology*, ed. Dunnette M. D. (Chicago, IL: Rand McNally). 1297–1343.

[B23] Lucas C. P. (2015). *A Promoção do Capital Psicológico, Work Engagement e Saúde Mental de Trabalhadores*. [Unpublished master dissertation]. Instituto Superior de Ciências Sociais e Políticas, Universidade de Lisboa, Lisboa.

[B24] Lucas H. M. Mónico L. S. Castro F. V. (2014). Psychological capital of individuals: what contributions for organizations? *INFAD Rev. Psicol.* 1 417–426. 10.17060/ijodaep.2014.n1.v5.701

[B25] Luthans F. Youssef C. M. (2004). Human, social, and now positive psychological capital management: investing in people for competitive advantage. *Manag. Depart. Facul. Publ.* 4 1–26.

[B26] Luthans F. Youssef-Morgan C. M. (2017). Psychological capital: an evidence-based positive approach. *Annu. Rev. Organ. Psychol. Organ. Behav.* 4 339–366. 10.1146/annurev-orgpsych-032516-113324

[B27] Luthans F. Avey J. Patera J. (2008). Experimental analysis of a web-based training intervention to develop positive psychological capital. *Acad. Manag. Learn. Educ.* 7 209–221. 10.5465/amle.2008.32712618

[B28] Luthans F. Avey J. Avolio B. Peterson S. (2010). The development and resulting performance impact of positive psychological capital. *Manag. Dep. Facul. Public.* 10:157. 10.1002/hrdq.20034

[B29] Luthans F. Avey J. Avolio B. Norman S. Combs G. (2006). Psychological capital development: toward a micro-intervention. *J. Organ. Behav.* 27 387–393. 10.1002/job.373

[B30] Luthans F. Avolio B. Avey J. Norman S. (2007a). Positive psychological capital: measurement and relationship with performance and satisfaction. *Leaders. Instit. Facul. Publicat.* 60 541–572. 10.1111/j.1744-6570.2007.00083.x

[B31] Luthans F. Luthans K. W. Luthans B. C. (2004). Positive psychological capital: beyond human and social capital. *Bus. Horiz.* 41 45–50. 10.1016/j.bushor.2003.11.007

[B32] Luthans F. Youssef C. M. Avolio B. J. (2007b). *Psychological Capital: Developing the Human Competitive Edge.* Oxford: Oxford University Press.

[B33] Luthans F. Youssef-Morgan C. M. Avolio B. J. (2015). *Psychological Capital and Beyond.* New York, NY: Oxford University Press.

[B34] MacDonald S. Maclntyrem P. (1997). The generic job satisfaction scale: scale development and its correlates. *Empl. Assist. Q.* 13 1–16. 10.1300/j022v13n02_01

[B35] Martinez L. Ferreira A. (2007). *Análise de Dados com SPSS.* Forte da Casa: Escolar Editora.

[B36] Neves A. (2016). *Capital Psicológico Positivo e Auto-Liderança: Um Estudo com Intervenção Formativa*. [Unpublished master dissertation]. Instituto Superior de Ciências Sociais e Políticas, Universidade de Lisboa, Lisboa.

[B37] Pini B. Mayes R. (2012). Gender, emotions and fly-in fly-out work. *Austr. J. Soc. Issue.* 47 71–86. 10.1002/j.1839-4655.2012.tb00235.x

[B38] Quivy R. Campenhoudt L. V. (1992). *Manual de Investigação em Ciências Sociais.* Lisboa: Gradiva.

[B39] Rego A. Marques C. Leal S. Sousa F. Cunha M. P. (2010). Psychological Capital and Performance of Civil Servants: exploring neutralizers in the context of an appraisal system. *Int. J. Hum. Resour. Manag.* 21:488459. 10.1080/09585192.2010.488459

[B40] Santos J. R. Mourão L. (2011). Impacto do treinamento como variável preditora da satisfação com o trabalho. *FEAUSP Rev. Administr.* 46 305–318. 10.5700/rausp1014

[B41] Serrão A. C. (2018). *Capital Psicológico Positivo: Um Estudo Sobre a Propagação e Contágio Entre Líderes e Liderados*. [Unpublished master dissertation]. Instituto Superior de Ciências Sociais e Políticas, Universidade de Lisboa, Lisboa.

[B42] Sibhoko O. (2017). *An investigation Into Employee Job Satisfaction and its Impacton Organizational Effectiveness*. Master thesis. Durban: Durban University of Technology.

[B43] Siqueira M. M. M. Padovam V. A. R. (2008). Bases teóricas de bem-estar subjetivo, bem-estar psicológico e bem-estar no trabalho. *Psicol. Teor. Pesqu.* 24 201–209. 10.1590/s0102-37722008000200010

[B44] Smith P. C. Kendall L. M. Hulin C. L. (1969). *The Measurement of Satisfaction in Work and Retirement*. Chicago, IL: Rand McNally.

[B45] Souza A. C. Milani D. Costa Alexandre N. M. (2015). Adaptação cultural de um instrumento para avaliar a satisfação no trabalho. *Rev. Brasil. Saúde Ocupac.* 15 219–227. 10.1590/0303-7657000113715

[B46] Spector P. E. (1985). Measurement of human services staff satisfaction: development of the job satisfaction survey. *Am. J. Commun. Psycol.* 13 693–713. 10.1007/bf00929796 4083275

[B47] Spector P. E. (1997). *Job satisfaction: Application, Assessment, Causes, and consequences*, Vol. 3. Thousand Oaks, CA: Sage.

[B48] Spector P. E. (2012). *Industrial and Organizational Psychology: Research and Practice.* Hoboken, NJ: John Wiley & Sons.

[B49] Spector P. E. (2006). *Industrial and Organizational Psychology: Research and Practice*. Hoboken, NJ: John Wiley & Sons Inc.

[B50] Torkington A. M. Larkins S. Gupta T. S. (2011). The psychosocial impacts of fly-in fly-out and drive-in drive-out mining on mining employees: a qualitative study. *Austr. J. Rural Health* 19 135–141. 10.1111/j.1440-1584.2011.01205.x 21605226

[B51] Weiss H. M. Brief A. P. (2001). *Affect at Work: A Historical Perspective, in Emotions at work: Theory, research, and applications for management*, eds Payne R. L. Cooper C. L. (Hoboken, NJ: John Wiley & Sons), 133–172.

[B52] Youssef-Morgan C. M. Luthans F. (2015). Psychological capital and well-being. *Stress Health* 31 180–188. 10.1002/smi.2623 26250352

